# RsWRKY15–*RsPDR12* module regulates Cd uptake and accumulation by promoting Cd efflux in radish (*Raphanus sativus* L.)

**DOI:** 10.1186/s43897-025-00195-7

**Published:** 2026-02-12

**Authors:** Shilin Ma, Zhihe Yang, Bingshuang Li, Liang Xu, Yan Wang, Yinbo Ma, Xiaoli Zhang, Jingxue Li, Kai Wang, Xinyu Zhang, Liwang Liu

**Affiliations:** 1 National Key Laboratory of Crop Genetics & Germplasm Enhancement and Utilization, Key Laboratory of Horticultural Crop Biology and Genetic Improvement (East China) of MOAR, Zhongshan Biological Breeding Laboratory, College of Horticulture, Nanjing Agricultural University, Nanjing, 211800 People’s Republic of China; 2 College of Horticulture and Landscape Architecture, Yangzhou University, Yangzhou, 225009 People’s Republic of China

**Keywords:** *Raphanus sativus*, Cd uptake and accumulation, RsPDR12, RsWRKY15, NMT

## Abstract

Cadmium (Cd), a non-essential and toxic heavy metal, accumulates in radish taproot and adversely affects radish safety and quality. Pleiotropic drug resistance (PDR) proteins play a crucial role in heavy metal uptake and accumulation in plants. However, the *PDR* gene-mediated regulation of Cd accumulation remains unexplored in radish. *RsPDR12* gene expression significantly increased under Cd stress. *RsPDR12* demonstrated Cd transport activity and decreased Cd accumulation in yeast cells, and its overexpression reduced root Cd concentration by Cd^2+^ efflux in *Arabidopsis*. *RsPDR12* alleviated Cd stress by enhancing membrane permeability and reactive oxygen species (ROS) scavenging in radish plants. RsWRKY15 was identified as the upstream regulatory factor of *RsPDR12*. Dual-luciferase assay demonstrated that RsWRKY15 bound to the *RsPDR12* promoter to activate its transcription. *RsWRKY15* expression and promoter activity were significantly induced under Cd stress. *RsWRKY15* overexpression mitigated oxidative damage and reduced root Cd concentration in radish and *Nicotiana benthamiana* plants, respectively. These findings advance understanding of the molecular mechanism underlying the RsWRKY15–*RsPDR12* mediated regulatory network of Cd accumulation and support the genetic improvement of low Cd-accumulation cultivars in radish breeding programs.

**Supplementary Information:**

The online version contains supplementary material available at 10.1186/s43897-025-00195-7.

## Core

Cd accumulates in the radish taproot, compromising the safe production of radish. RsPDR12 demonstrated significant Cd transport activity and reduced Cd accumulation in yeast cells. *RsPDR12* overexpression decreased Cd accumulation by mediating Cd^2+^ efflux in roots. RsWRKY15 binds to the *RsPDR12* promoter to activate its transcription. *RsWRKY15* overexpression reduces Cd accumulation by decreasing Cd^2+^ influx in roots under Cd Stress. The RsWRKY15–*RsPDR12* module regulates Cd accumulation by promoting Cd efflux in roots.

## Gene & accession numbers

Information of genes in this study can be found in the database (https://bigd.big.ac.cn) under the accession numbers: *RsPDR12*(Rsa1g010600); *RsWRKY15*(Rsa4g037000).

## Introduction

Cadmium (Cd) is one of the most toxic heavy metal (HM) pollutants due to its long half-life and non-biodegradability. Its pervasive toxicity affects various tissues and organs in plants, animals and humans (Sheng et al. 2019; McLaughlin et al. 2021; Tang et al. 2024). Cd contamination has become a significant problem, severely impacting agricultural productivity by industrial and natural factors (Zhao et al. 2021; Yokosho et al. 2021). Under Cd stress, reactive oxygen species (ROS) are triggered in plants, resulting in cellular oxidative damage and disruption of physiological and biochemical processes (Song et al. 2017; Lu et al. 2020). Consequently, plants exhibit restricted growth, decreased photosynthetic rates, impaired respiration, leaf yellowing, inhibited root growth, and potential mortality (Han et al. 2019; Zhao et al. 2021). Understanding the molecular mechanisms of HM accumulation in plants is therefore essential for mitigating HM stress.

Under HM stress, specific transporters including ATP-binding cassettes (ABCs), P1B ATPases, and natural resistance–associated macrophage proteins (NRAMPs) respond and perform crucial functions in HM uptake, accumulation, and translocation (Ziegler et al. 2017; Liu et al. 2020; Ashraf et al. 2021; Zheng et al. 2020). ABC transporters function as essential membrane transport proteins (Kuromori et al. 2017; Gani et al. 2021). Pleiotropic drug resistance (PDRs) proteins, members of the ABCG subfamily of the ABC family, transport HMs and other substrates, including phytohormones and lipids (Lefèvre et al. 2018; Xie et al. 2021; Zhang et al. 2020). Compared with wild-type (WT) plants, *AtPDR8*-overexpressing (OE) *Arabidopsis* exhibited reduced Cd accumulation and decreased Cd content, while Cd accumulation increased in *pdr8* mutant plants (Kim et al. 2007). Lead (Pb) accumulation in *Arabidopsis* is regulated by the expression of *AtPDR12* and other enzyme-related genes (Fan et al. 2016). The *Arabidopsis pdr12* mutant shows sensitivity to Pb, while *AtPDR12* overexpression reduces Pb accumulation in plants (Lee et al. 2005). However, the molecular mechanism by which PDRs influence HM accumulation in radish taproots remains unclear.

Transcription factors (TFs), including MYBs, WRKYs, bZIPs, and HSFs, are essential components of the gene expression regulatory network under HM stress (Agarwal et al. 2006; Zhang et al. 2019, 2023a; Tang et al. 2019, 2024; Sheng et al. 2019; Han et al. 2019). WRKYs, containing one or two conserved WRKY domains—comprising the WRKYGQK core sequence and a C_2_H_2_- or C_2_HC-type zinc finger motif in the N-terminal—constitute one of the largest TF families in higher plants (Rushton et al. 2010). WRKY TFs regulate plant responses to biotic and abiotic stresses by activating or inhibiting the transcription of downstream functional genes by binding to the W-box [(T)(T)TGAC(C/T)] element in their target genes promoter sequences (Han et al. 2019). In *Arabidopsis*, Cd accumulation decreased with *AtWRKY13* overexpression, while increased in the *AtWRKY13* loss-of-function mutant. ChIP-qPCR and yeast one-hybrid (Y1H) assays demonstrated that AtWRKY13 binds to the W-box region in the *AtPDR8* gene promoter, facilitating Cd^2+^ removal from the cytoplasm by activating *AtPDR8* transcription and positively regulating plant Cd accumulation (Sheng et al. 2019). Conversely, maize ZmWRKY4 mediates the Cd stress response by positively regulating the expression of *ZmSOD4* and *ZmAPX* genes (Hong et al. 2017).

Radish (*Raphanus sativus* L.), a member of the Brassicaceae family, is an economically significant root vegetable crop. Under Cd stress, the radish taproot accumulates more Cd compared with other organs (Xu et al. 2012; Sheng et al. 2019). Because the taproot constitutes the main edible portion of radish, Cd accumulation presents a potential risk to human health by food chain contamination (Zhao and Wang 2020). *PDR* genes play a crucial role in plant HM absorption and accumulation. Although PDR gene family members can reduce Cd content under Cd stress, the biological function and regulatory mechanism governing Cd uptake and accumulation in radish remain unclear. Pb treatment can alter the methylation levels of *RsPDR12* in radish, affecting its expression (Tang et al. 2021). Previous transcriptome data revealed significant induction of *RsPDR12* gene expression under Cd stress (Zhang et al. 2023b). We evaluated *RsPDR12* expression under Cd stress by Reverse Transcription quantitative Polymerase Chain Reaction (RT-qPCR) analysis and investigated its function in yeast, *Arabidopsis*, and radish. The upstream TF RsWRKY15, which regulates *RsPDR12*, was identified using a Y1H library to examine the transcriptional regulatory network of the TF–*RsPDR12* module under Cd stress. In addition, *RsWRKY15* function under Cd stress was characterized in *N. benthamiana* and radish, leading to the proposal of the RsWRKY15–*RsPDR12* module as a regulator of Cd uptake and accumulation in radish taproots. These findings illuminate the PDR-mediated regulatory network under Cd stress in radish, contributing to Cd uptake and accumulation and supporting the development of low Cd-accumulation cultivars in radish breeding programs.

## Results

### Expression profiling and promoter activity analysis of *RsPDR12* under Cd treatment

**Fig. 1:**
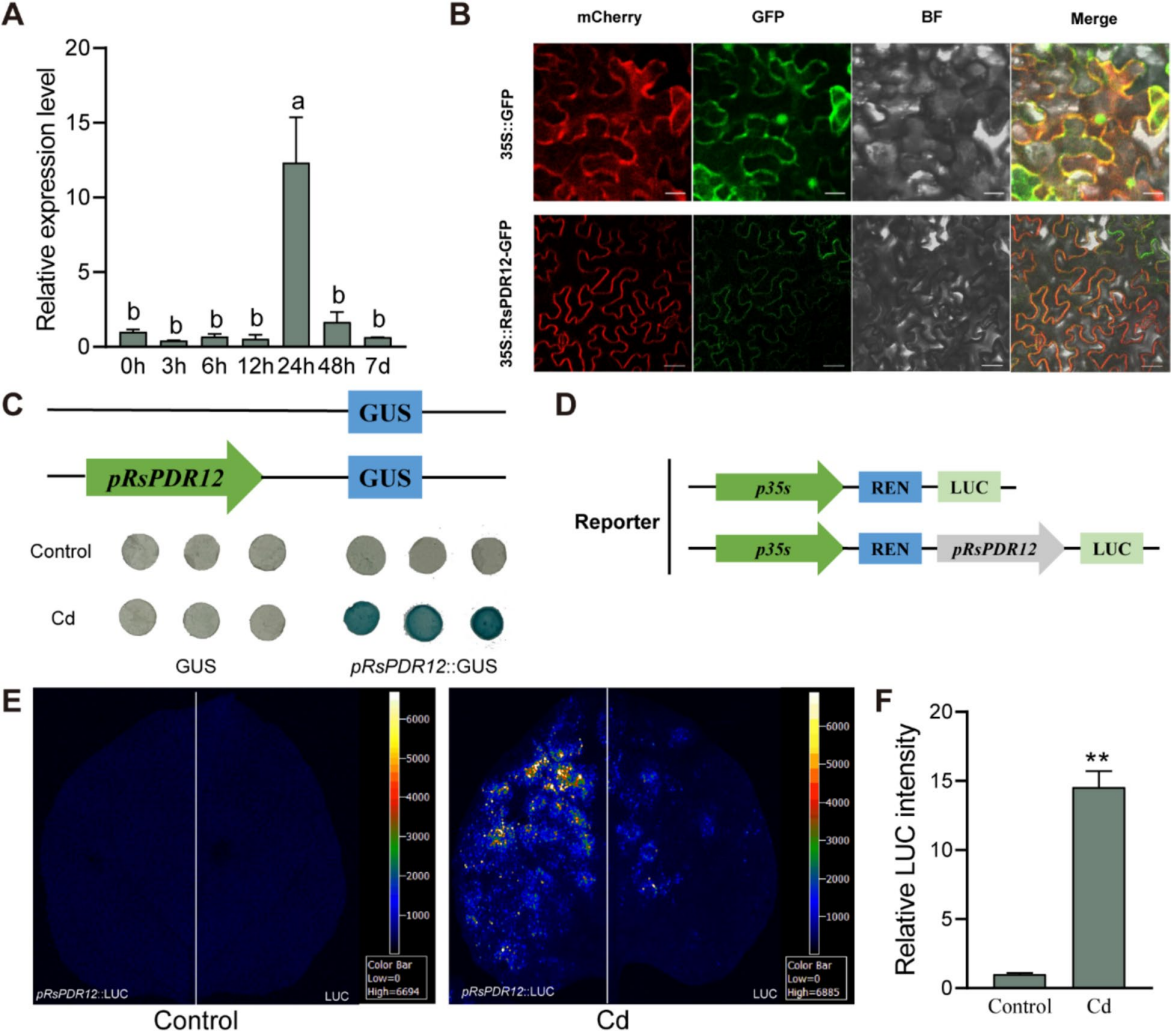
Characterization and promoter activity analysis of *RsPDR12*. **A** Expression profiling of the *RsPDR12* gene in radish roots under Cd treatment (0.1 mM CdCl_2_). Different letters above the bars indicate significant differences (*P* < 0.05). **B** The subcellular localization of *RsPDR12* in *N. benthamiana* leaves. The PM-mCherry was used as a membrane marker. Scale bar = 50 µm. **C** GUS staining analysis of *pRsPDR12* in *N. benthamiana* leaves under Cd treatment (0.27 mM CdCl_2_, 8 h). **D** Structures of the report vector. **E** LUC assay of *pRsPDR12* in *N. benthamiana* leaves under Cd treatment (0.27 mM CdCl_2_, 8 h). **F** Relative LUC intensity of the *pRsPDR12* promoter under Cd treatment. Data are the mean ± SD of three replicates (*t*-test; **P < 0.01)

Consistent with transcriptome data, RT-qPCR analysis revealed that *RsPDR12* expression levels in the radish taproot were induced and peaked at 24 h under Cd treatment (Fig. 1A). The coding sequence (CDS) of *RsPDR12* comprised 4245 bp and encoded 1414 amino acids, exhibiting 93.53% similarity with AtPDR12 (Fig. S1). Subcellular localization analysis revealed that the GFP-tagged RsPDR12 protein localized to the plasma membrane (Fig. 1B).

The *RsPDR12* promoter-driven GUS activity demonstrated greater intensity compared with control under Cd treatment (Fig. 1C). The luciferase (LUC) signal of the *RsPDR12* promoter increased upon leaf exposure to Cd treatment (Fig. 1D and E), and the relative LUC intensity of the *RsPDR12* promoter showed significant enhancement (Fig. 1F), indicating marked activation of *RsPDR12* promoter activity under Cd stress.

### Cd transport activity of *RsPDR12* in yeast cells

**Fig. 2:**
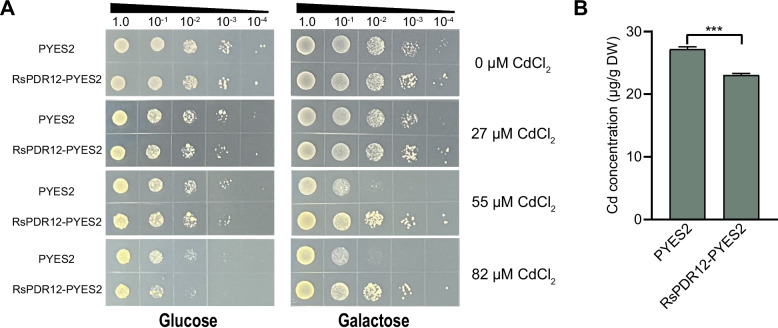
Transport ability of the *RsPDR12* gene under Cd treatment. **A** Dot assay of yeast cells expressing RsPDR12-pYES2 or the empty vector (pYES2) on medium containing glucose, galactose, and Cd. **B** Cd concentrations in yeast expressing *RsPDR12* under Cd treatment (0.27 mM CdCl_2_) (*t*-test; ***P < 0.001)

To investigate the Cd transport activity of *RsPDR12* in yeast, the empty vector and RsPDR12-pYES2 were transformed into yeast strain BY4741. In the presence of the transcription inhibitor glucose, no significant growth differences were observed between yeast cells containing the empty vector and those expressing *RsPDR12*, and growth was inhibited as the CdCl_2_ concentration increased (Fig. 2A). In the presence of the transcription inducer galactose, no significant growth differences were observed between yeast cells with pYES2 and RsPDR12-pYES2 under normal conditions. However, under 55 and 82 μM CdCl₂ treatments, yeast cells carrying the empty vector exhibited significantly inhibited growth compared with those expressing *RsPDR12* (Fig. 2A). The Cd content was lower in yeast cells expressing *RsPDR12* than in cells with the empty vector after 0.27 mM CdCl_2_ treatment for 12 h (Fig. 2B), demonstrating that *RsPDR12* exhibits Cd transport activity and decreases Cd accumulation in yeast.

### *RsPDR12* overexpression reduces Cd accumulation

**Fig. 3:**
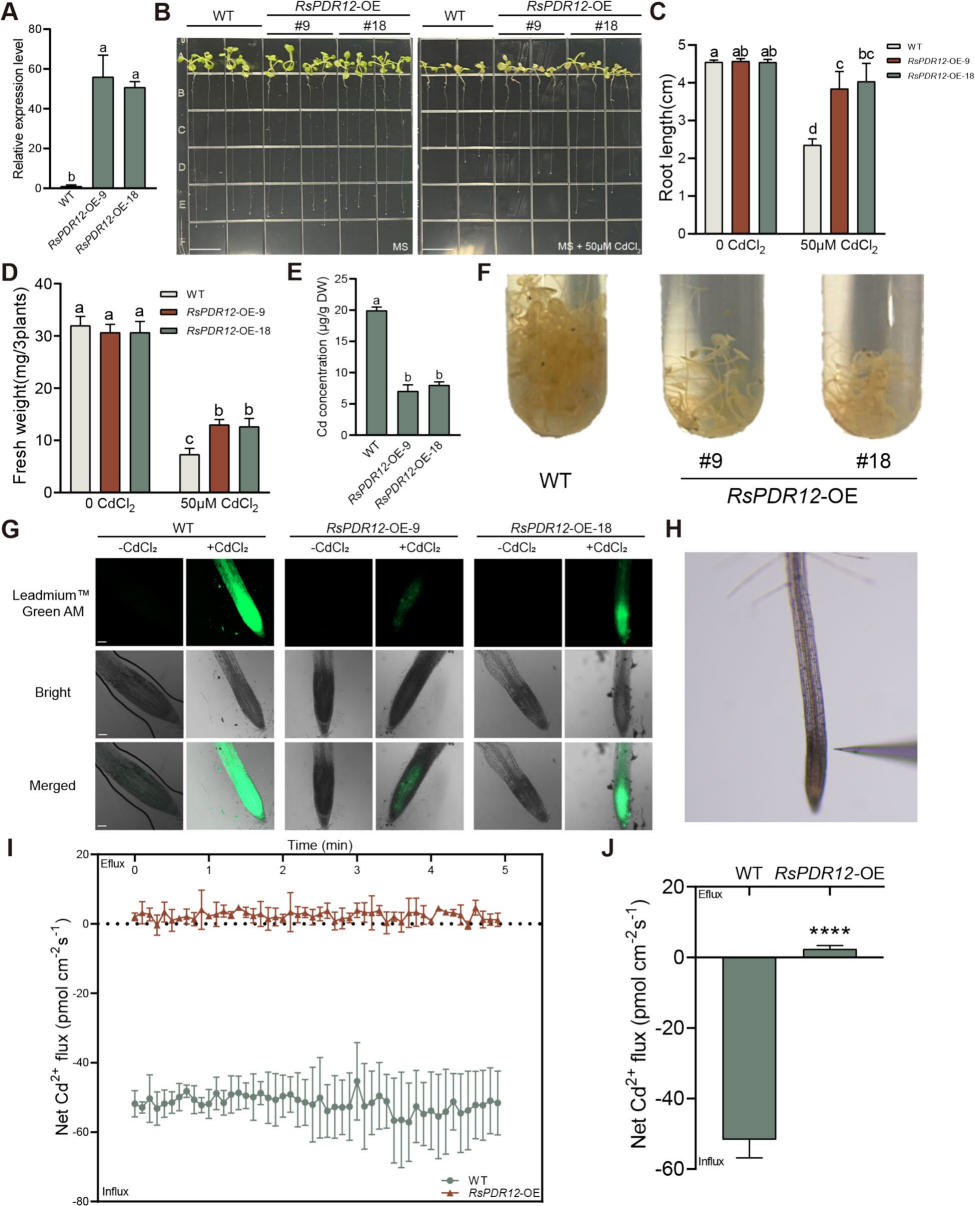
*RsPDR12* reduces Cd accumulation in *Arabidopsis* plants. **A** Expression level of *RsPDR12* in WT and *RsPDR12*-OE lines. **B** Phenotype changes in WT and *RsPDR12*-OE lines under Cd treatment (50 μM CdCl_2_). Bar = 1 cm. **C** Changes in the root length and (**D**) fresh weight. **E** Cd content of WT and *RsPDR12*-OE lines under Cd treatment (50 μM CdCl_2_). Data are presented as the mean ± SD, *n* = 3. Bars with different lowercase letters are significantly different at *P* < 0.05. **F** Dithizone staining of WT and *RsPDR12*-OE lines under Cd treatment (50 μM CdCl_2_). **G** Cadmium content detection of *RsPDR12*-overexpressing *Arabidopsis* lines with Cd-fluorescent analysis. Bar = 50 μm. **H** Testing position of the probe in the roots. **I** Transient Cd^2+^ fluxes measured every 6 s in 14-d-old seedlings of *RsPDR12*-overexpressing *Arabidopsis* and WT exposed to 0.1 mM Cd for 8 h. **J** Average Cd^2+^ fluxes for 5 min. Data are the mean ± SD (*n* = 5). Bars indicate significant differences between the roots of *RsPDR12*-OE *Arabidopsis* and WT at ****P < 0.0001

To examine the function of *RsPDR12* in *Arabidopsis* under Cd stress, the overexpression lines *RsPDR12*-OE-9 and *RsPDR12*-OE-18 were selected for further analysis (Fig. 3A). Under normal conditions, both *RsPDR12*-OE lines and WT plants demonstrated normal growth on Murashige and Skoog (MS) plates. However, under Cd treatment, the *RsPDR12*-OE-9 and *RsPDR12*-OE-18 lines displayed longer root length, higher fresh weight, and lower Cd content compared with WT (Fig. 3B–E). Dithizone staining revealed that *RsPDR12*-OE-9 and *RsPDR12*-OE-18 lines exhibited fewer red–black Cd–dithizone precipitates than WT under Cd treatment (Fig. 3F). Thus, *RsPDR12* overexpression enhances growth and reduces Cd accumulation in *Arabidopsis*.

The Cd-fluorescent probe assay revealed lower fluorescence in the root systems of *RsPDR12*-OE lines compared with WT (Fig. 3G), indicating reduced Cd concentration in *RsPDR12*-OE lines. In addition, non-invasive micro-test technology (NMT) measured the dynamic changes in Cd^2+^ fluxes in the root elongation zone (Fig. 3H). The transient and average Cd^2+^ fluxes in *RsPDR12*-OE roots were positive (efflux), while those in WT roots were negative (influx), indicating that *RsPDR12* promotes Cd^2+^ efflux from plant roots under Cd treatment (Fig. 3I and J). These results suggest that *RsPDR12* reduces Cd uptake and accumulation in plants by facilitating Cd^2+^ efflux under Cd stress.

### ROS accumulation was reduced in *RsPDR12-*OE radish

**Fig. 4:**
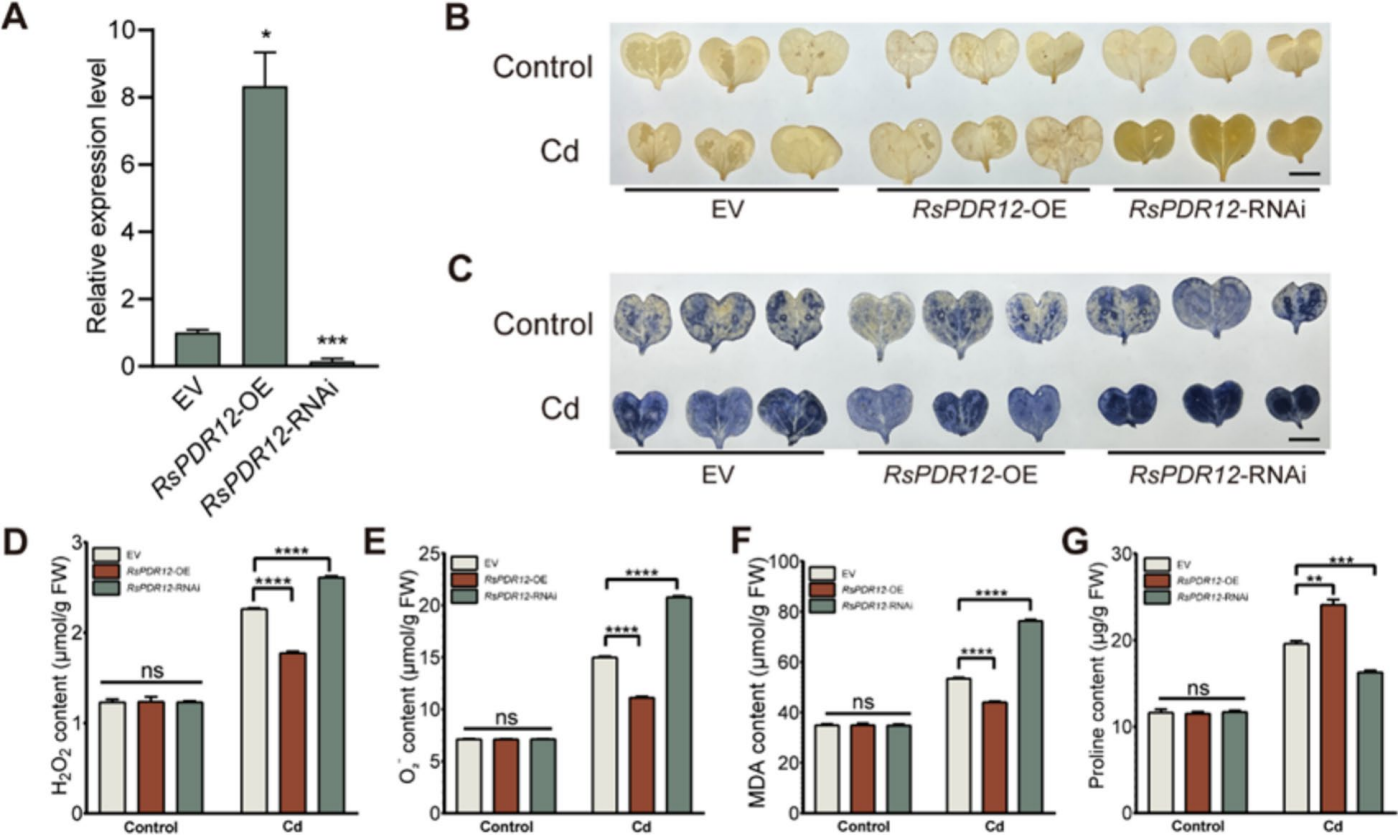
ROS accumulation was reduced in *RsPDR12*-overexpressing radish. **A** Expression profile of *RsPDR12* in *RsPDR12*-OE and *RsPDR12*-RNAi radish cotyledons. **B** DAB and (**C**) NBT staining of radish cotyledons transiently infected with EV, *RsPDR12*-OE, and *RsPDR12*-RNAi under control and Cd stress (0.27 mM CdCl_2_, 8 h). Scale bar = 1 cm. **D** H_2_O_2_, **E** O_2_.^−^, **F** MDA, and (**G**) proline content in EV, *RsPDR12*-OE, and *RsPDR12*-RNAi radish plants under control conditions and Cd stress (0.27 mM CdCl_2_, 8 h). Data are the mean ± SD of three replicates (*t*-test; ** *P* < 0.01; *** *P* < 0.001; ****P < 0.0001; ns, not significant)

*RsPDR12* was transiently overexpressed and silenced in radish cotyledons (Fig. 4A). 3,3′-Diaminobenzidine (DAB) and nitroblue tetrazolium (NBT) staining showed no significant differences among EV, *RsPDR12*-OE, and *RsPDR12*-RNAi cotyledons under normal conditions (Fig. 4B and C). Under Cd stress, *RsPDR12*-OE cotyledons displayed lighter histochemical staining compared with EV cotyledons, whereas *RsPDR12*-RNAi cotyledons exhibited deeper staining than EV cotyledons (Fig. 4B and C).

The levels of hydrogen peroxide (H_2_O_2_), superoxide (O_2_^−^), malondialdehyde (MDA) and proline were not significantly different among EV, *RsPDR12*-OE and *RsPDR12*-RNAi cotyledons under normal conditions (Fig. 4D–G). However, under Cd treatment, the levels of H_2_O_2_, O_2_^−^, and MDA in *RsPDR12*-OE cotyledons were lower than those in EV cotyledons, while they were significantly elevated in *RsPDR12*-RNAi cotyledons (Fig. 4D–F). Conversely, proline content was significantly increased in *RsPDR12*-OE cotyledons and decreased in *RsPDR12*-RNAi cotyledons under Cd stress (Fig. 4G).

These results demonstrate that *RsPDR12* overexpression reduces ROS accumulation and membrane permeability, presumably due to decreased Cd uptake and accumulation under Cd stress in radish.

### RsWRKY15 binds to the *RsPDR12* promoter and activates its transcription

**Fig. 5:**
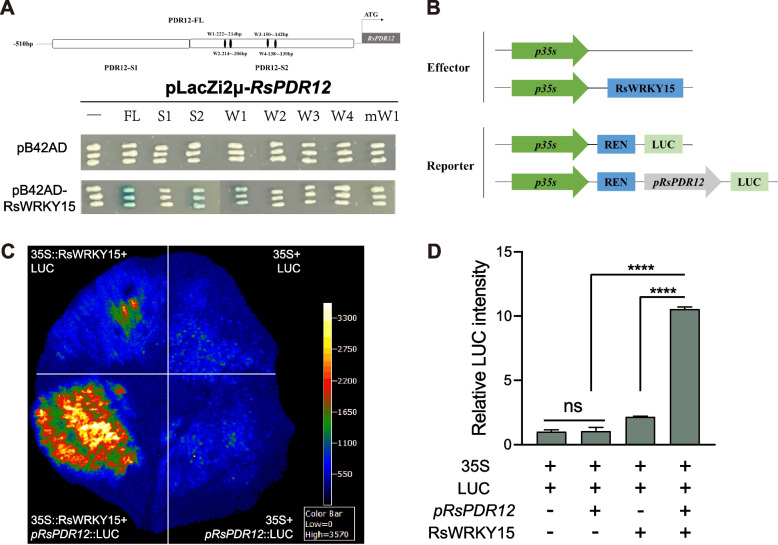
RsWRKY15 activates *RsPDR12* transcription by directly binding to its promoter. **A** RsWRKY15 binds to the W1 motifs of *RsPDR12* promoter in a Y1H assay. **B** Generation of the effector and reporter constructs for DLA. **C** Transient expression assays of the empty vector, RsWRKY15, and *RsPDR12* promoter in *N. benthamiana* leaves. **D** Relative LUC intensity from the empty vector, RsWRKY15, and *RsPDR12* promoter. Data are the mean ± SD of three replicates (*t*-test; **** *P* < 0.0001; ns, not significant)

To investigate the regulation of *RsPDR12* under Cd stress, a 510-bp upstream promoter sequence of *RsPDR12* was isolated, and the direct upstream activator was identified using Y1H screening against a radish cDNA library (Fig. S2). The TF RsWRKY15 was identified by the Y1H library approach. The Y1H assay subsequently confirmed RsWRKY15 binding to the FL and S2 fragments of the *RsPDR12* promoter. Notably, three tandem repeats of the W-box (W1; core sequence: GAGTCACA) significantly activated the lacZ reporter gene. However, the interaction ceased when the W-box sequence was altered from GTCA to ATCC, indicating that RsWRKY15 functions as an upstream regulator of *RsPDR12* by directly binding to the specific W1 in its promoter region (Fig. 5A). DLA results demonstrated that the LUC signal and activity significantly increased when *35S::RsWRKY15* was co-expressed with *pRsPDR12*::LUC (Fig. 5C and D), confirming RsWRKY15 as an upstream TF regulating *RsPDR12* gene expression.

### Expression profiling and promoter activity of the *RsWRKY15* gene under Cd treatment

**Fig. 6:**
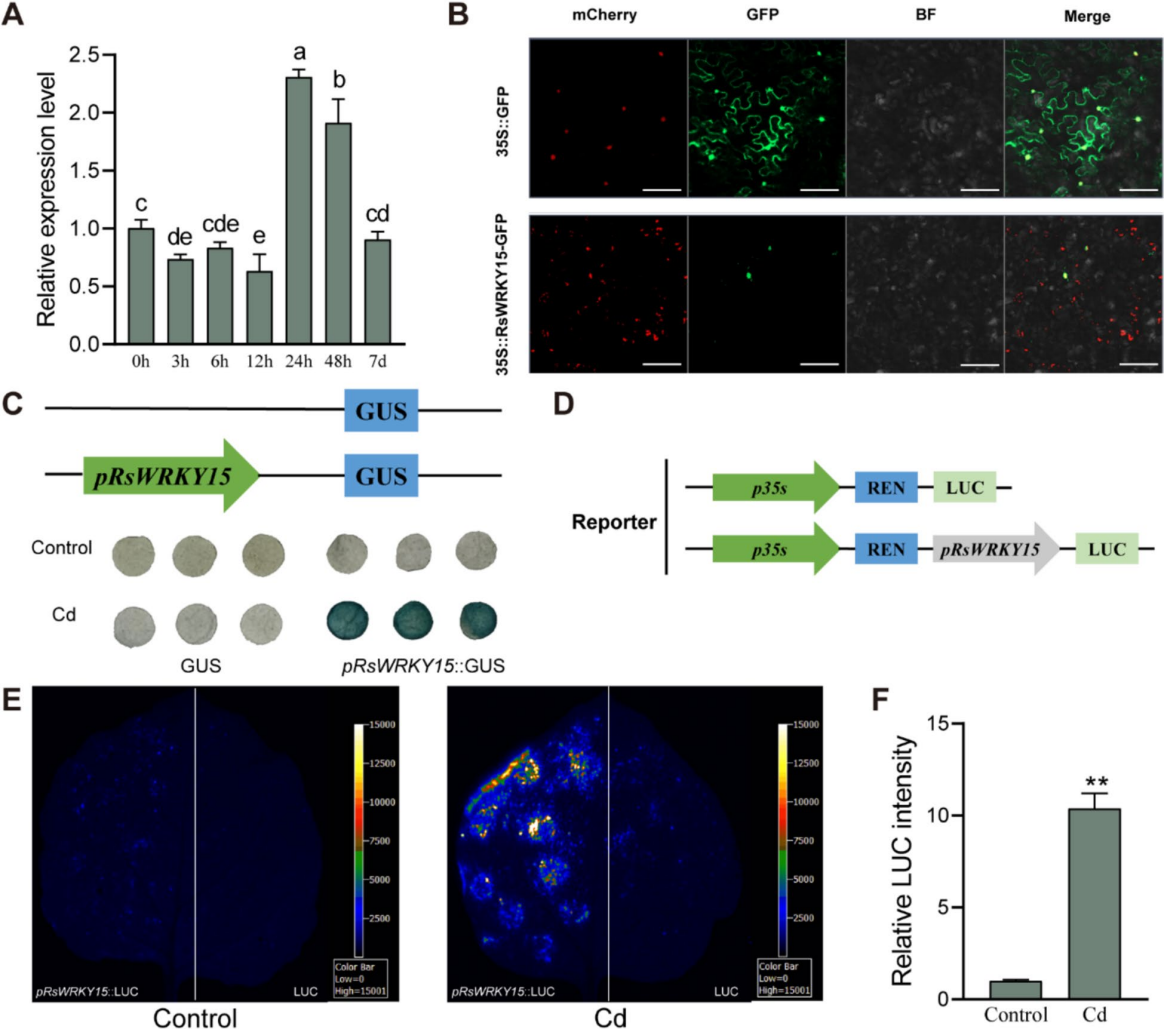
Characterization and differential expression profile of *RsWRKY15*. **A** Expression profiling of the *RsWRKY15* gene in radish roots under Cd treatment (0.1 mM CdCl_2_). Different letters above the bars indicate significant differences (*P* < 0.05). **B** Subcellular localization of RsWRKY15 in *N. benthamiana* leaves. pSuper::NF-YA4-mCherry was used as a nuclear marker. Scale bar = 50 µm. **C** GUS staining analysis of *pRsWRKY15* in *N. benthamiana* under control conditions and Cd treatment (0.27 mM CdCl_2_, 8 h). **D** Structure of the report vector. **E** LUC assay of *pRsWRKY15* in *N. benthamiana* leaves under control conditions and Cd treatment (0.27 mM CdCl_2_, 8 h). **F** Relative LUC intensity of the *pRsWRKY15* promoter under Cd treatment. Data are the mean ± SD of three replicates (*t*-test; **P < 0.01)

The complete CDS of *RsWRKY15* is 963 bp and encodes 320 amino acids, containing a conserved WRKYGQK domain and a C_2_H_2_ zinc finger motif at the C terminus (Fig. S3), both characteristic features of the WRKY TF family. RT-qPCR analysis revealed that the *RsWRKY15* expression level reached its maximum in radish at 24 h under Cd treatment (Fig. 6A). Subcellular localization analysis demonstrated that the RsWRKY15 protein was localized in the nucleus (Fig. 6B).

The GUS activity of *RsWRKY15* exhibited greater intensity compared with control under Cd treatment (Fig. 6C). The LUC signal and relative intensity of leaves containing *pRsWRKY15*::LUC increased following Cd exposure, demonstrating that the promoter activity of *RsWRKY15* was strongly induced by Cd stress (Fig. 6D–F).

### *RsWRKY15* overexpression reduces Cd accumulation in *N. benthamiana* plants

**Fig. 7:**
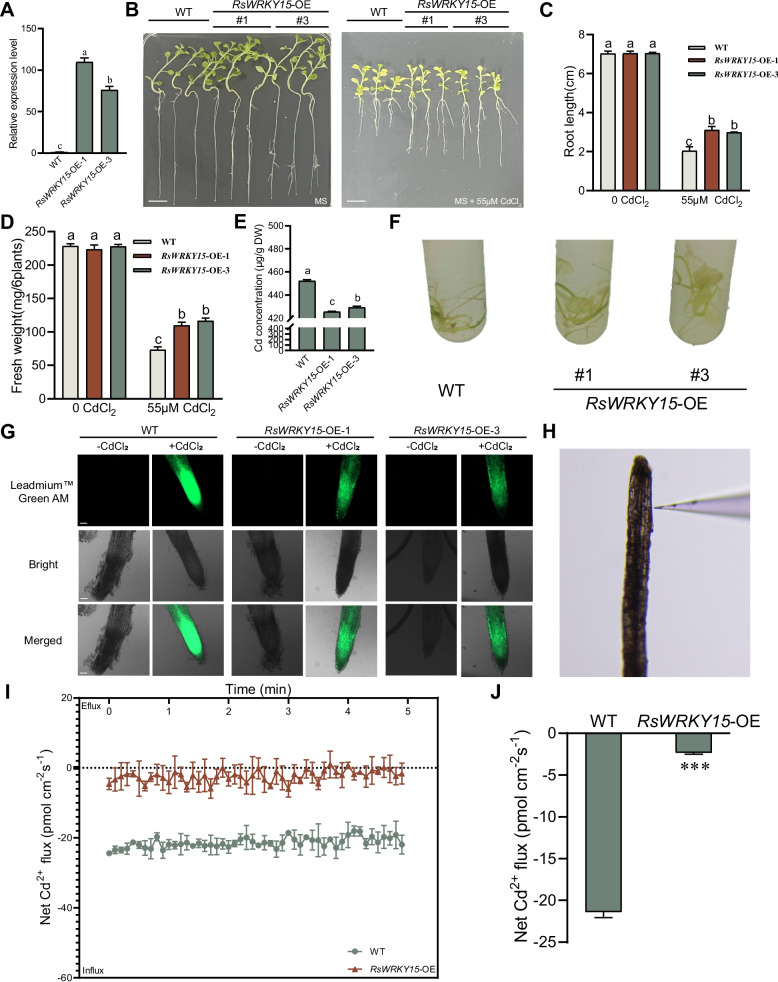
*RsWRKY15* reduces Cd accumulation in *N. benthamiana* plants. **A** Expression level of *RsWRKY15* in WT and *RsWRKY15*-OE lines. **B** Phenotype changes under Cd treatment (55 μM CdCl_2_). Bar = 1 cm. **C** Root length. **D** Fresh weight. **E** Cd content under Cd treatment (55 μM CdCl_2_). Data are presented as the mean ± SD, *n* = 3. Bars with different lowercase letters are significantly different at *P* < 0.05. **F** Dithizone staining under Cd treatment (55 μM CdCl_2_). **G** Cd content detection with Cd fluorescence. Bar = 50 μm. **H** Testing position of the probe in roots. **I** Transient Cd^2+^ fluxes measured every 6 s in 21-d-old seedlings of *RsWRKY15*-OE lines and WT under Cd treatment (0.1 mM Cd for 8 h). **J** Average Cd^2+^ fluxes in 5 min. Data are the mean ± SD (*n* = 5). Asterisks indicate significant differences between the roots of *RsWRKY15*-OE and WT at *P* < 0.001

To investigate the function of *RsWRKY15* under Cd treatment, overexpression lines *RsWRKY15*-OE-1 and *RsWRKY15*-OE-3 were selected for functional analysis (Fig. 7A). Under normal conditions, both *RsWRKY15*-OE lines and WT exhibited normal growth on MS plates, while the *RsWRKY15*-OE-1 and *RsWRKY15*-OE-3 lines demonstrated longer roots, higher fresh weight, and lower Cd content than WT plants under Cd treatment (Fig. 7B–E). Dithizone staining revealed that *RsWRKY15*-OE lines contained fewer red–black Cd–dithizone precipitates (Fig. 7F), indicating that *RsWRKY15* overexpression enhanced growth vigor and reduced Cd accumulation.

Cd fluorescence probe staining analysis results showed reduced fluorescence in the root systems of *RsWRKY15*-OE lines compared with WT, indicating decreased Cd concentration in *RsWRKY15*-OE lines (Fig. 7G). Furthermore, NMT measured the dynamic changes in Cd^2+^ fluxes in the root elongation zone (Fig. 7H). The transient and average Cd^2+^ fluxes in roots of *RsWRKY15*-OE lines decreased under Cd treatment compared with WT (Fig. 7I and J), suggesting that *RsWRKY15* reduces Cd accumulation in plants by decreasing Cd^2+^ influx under Cd stress.

The *NtPDR12* expression level was significantly upregulated, exhibiting a 6.09-fold increase compared with WT (Fig. S4).

### ROS accumulation is reduced in *RsWRKY15*-OE radish

**Fig. 8:**
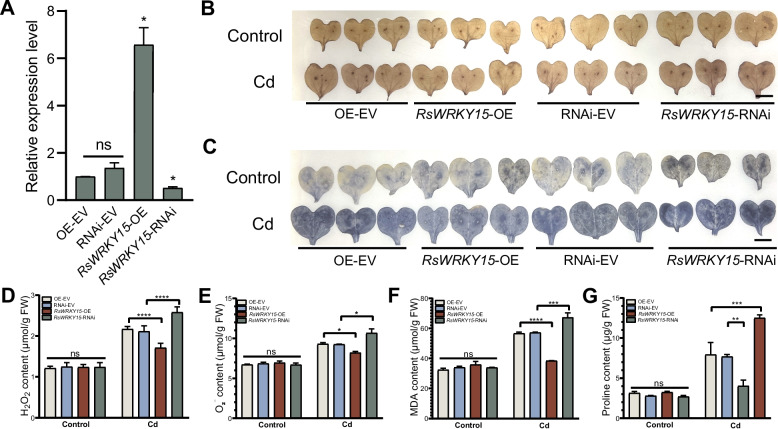
ROS accumulation was reduced in *RsWRKY15*-overexpressing radish. **A** *RsWRKY15* expression in OE-EV, RNAi-EV, *RsWRKY15*-OE, and *RsWRKY15*-RNAi radish cotyledons. **B** DAB and (**C**) NBT staining of radish cotyledons transiently transformed with the OE-EV, RNAi-EV, *RsWRKY15*-OE, and *RsWRKY15*-RNAi under Cd stress (0.27 mM CdCl_2_, 8 h). Scale bar = 1 cm. **D** H_2_O_2_, **E** O_2_.^−^, **F** MDA and (**G**) proline contents, respectively, in OE-EV, RNAi-EV, *RsWRKY15*-OE, and *RsWRKY15*-RNAi radish plants under Cd stress (0.27 mM CdCl_2_, 8 h). Data are the mean ± SD of three replicates (*t*-test; * *P* < 0.05; ** *P* < 0.01; *** *P* < 0.001; ****P < 0.0001; ns, not significant)

*RsWRKY15* underwent transient OE and silencing in radish cotyledons (Fig. 8A). The Cd content significantly decreased in *RsWRKY15*-OE and increased in *RsWRKY15*-RNAi compared with EV in radish cotyledons (Fig. S5). DAB and NBT staining revealed no significant differences between OE-EV, *RsWRKY15*-OE, RNAi-EV, and *RsWRKY15*-RNAi cotyledons under normal conditions (Fig. 8B and C). Under Cd stress, *RsWRKY15*-OE cotyledons displayed lighter histochemical staining compared with OE-EV cotyledons, while RsWRKY15-RNAi cotyledons exhibited deeper histochemical staining compared with RNAi-EV cotyledons (Fig. 8B and C).

Under normal conditions, no significant changes were observed in the levels of H_2_O_2_, O_2_^−^, MDA and proline between *RsWRKY15*-OE and OE-EV, and *RsWRKY15*-RNAi and RNAi-EV cotyledons (Fig. 8D–G). However, under Cd treatment, *RsWRKY15*-OE cotyledons showed lower levels of H_2_O_2_, O_2_^−^, and MDA than OE-EV cotyledons, while these levels significantly increased in *RsWRKY15*-RNAi cotyledons compared with RNAi-EV cotyledons (Fig. 8D–F). Conversely, under Cd stress, proline content was notably higher in *RsWRKY15*-OE cotyledons and reduced in *RsWRKY15*-RNAi cotyledons compared with OE-EV and RNAi-EV cotyledons (Fig. 8G). *RsPDR12* expression was elevated in *RsWRKY15*-OE cotyledons compared with OE-EV cotyledons, while showing significant reduction in *RsWRKY15*-RNAi cotyledons compared with RNAi-EV cotyledons (Fig. S6).

*RsWRKY15* overexpression can reduce ROS accumulation, likely by decreased Cd uptake and accumulation under Cd stress in radish.

## Discussion

### *RsPDR12* is a vital participant in reducing Cd accumulation under Cd stress

Stress induced by Cd, Pb, and other HMs is an increasingly significant environmental concern, affecting both plant productivity and growth potential while presenting serious risks to human health (Matsuda et al. 2009; Zhao et al. 2021; Chen et al. 2024; Guo et al. 2024; Wang et al. 2024a, b). Several ABCG subfamily transporters—including AtABCG36/AtPDR8, RsABCG36/RsPDR8, OsABCG40/OsPDR9, AtABCG40/AtPDR12, AhPDR33, and OsPDR20—are potential mediators in plant responses to HM stress (Fan et al. 2016; Ziegler et al. 2017; Sheng et al. 2019; Gupta et al. 2019; Ashraf et al. 2021; Zhang et al. 2023b; Wang et al. 2024; Li et al. 2024). The Cd efflux pumps, *AtABCG36*/*AtPDR8* and *RsABCG36*/*RsPDR8* demonstrate capacity to reduce Cd accumulation in *Arabidopsis* and radish (Kim et al. 2007; Sheng et al. 2019; Zhang et al. 2023b). *RsPDR8* expression shows dramatic upregulation under both Cd and Pb stresses (Zhang et al. 2023b). In this investigation, *RsPDR12* expression increased under Cd treatment in radish, with *RsPDR12* localizing to the plasma membrane (Fig. 1). Given that *AtPDR12* similarly localizes to the plasma membrane and responds to Pb stress, *RsPDR12* likely plays a comparable positive role in managing Cd stress. *OsABCG36*/*OsPDR9* functions as a Cd efflux pump, transporting Cd from rice root cells, as demonstrated by heterologous yeast and genetic transformation in rice (Fu et al. 2019). In addition, yeast cells transformed with *RsPDR12* exhibited enhanced growth and decreased Cd content compared with cells containing the empty vector (Fig. 2). While *AtPDR8* overexpression in *Arabidopsis* reduces Cd accumulation (Kim et al. 2007), *AtPDR12* overexpression decreases Pb accumulation by regulating enzyme-related genes (Lee et al. 2005; Fan et al. 2016). In the present study, *RsPDR12*-OE lines demonstrated more vigorous growth and significantly reduced Cd content compared with WT plants under Cd treatment, indicating that *RsPDR12* overexpression reduces Cd accumulation and enhances Cd^2+^ efflux under Cd stress compared with WT plants (Fig. 3).

### TF RsWRKY15 reduces Cd accumulation in plants

In plants, TFs mediate various metal stress perception and coping mechanisms (Liu et al. 2017; Zhu et al. 2018; Li et al. 2023). The WRKY TF family represents one of the most significant regulatory families involved in HM stress responses (Hong et al. 2017; Sheng et al. 2019; Han et al. 2019; Cai et al. 2020; Jia et al 2021; Li et al. 2023; Zhang et al. 2023a). In *Arabidopsis*, overexpression and loss-of-function of *AtWRKY12* correlated with increased and decreased Cd accumulation by the phytochelatin synthesis pathway (Han et al. 2019). *AtWRKY13* overexpression decreased Cd accumulation, while loss-of-function of *AtWRKY13* resulted in increased Cd accumulation and sensitivity in *Arabidopsis* (Sheng et al. 2019). *AtWRKY33* responds to Cd stress and effectively reduces Cd accumulation (Zhang et al. 2023a). Similarly, *AtWRKY45* diminishes Cd accumulation (Li et al. 2023). *ZmWRKY4* appears to regulate the expression of *ZmSOD4* and *ZmAPX* genes under Cd stress (Hong et al. 2017). *GmWRKY142* overexpression decreased Cd uptake and accumulation in soybean (Cai et al. 2020), while *GmWRKY172* overexpression increased Cd accumulation and reduced seed Cd content (Xian et al. 2023). *TaWRKY70* overexpression decreased Cd accumulation in wheat (Jia et al 2021). *PyWRKY75* overexpression enhanced Cd accumulation and chlorophyll content of poplar (Wu et al. 2022). These findings indicate that WRKY-mediated responses to Cd stress are conserved across plant species. Although, evidence regarding the potential role of WRKYs in Cd stress response remains limited in radish, *CmWRKY15-1* is involved in biotic stress resistance in chrysanthemum (Chen et al. 2023). This study initially identified the function of *RsWRKY15* in facilitating decreased Cd accumulation under Cd stress in radish. *RsWRKY15* expression increased under Cd treatment, and *RsWRKY15*-OE lines exhibited more robust growth and significantly lower Cd content under Cd stress. NMT demonstrated that *RsWRKY15* overexpression reduced Cd accumulation in plants by decreasing Cd^2+^ influx under Cd stress. These results suggest that *RsWRKY15* plays a positive role in managing Cd stress in plants.

### RsWRKY15–*RsPDR12* reduces Cd accumulation

**Fig. 9:**
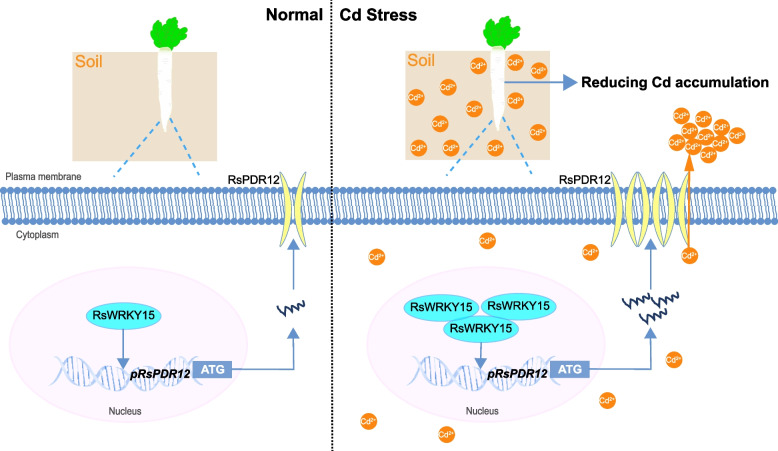
Putative model of Cd uptake and accumulation regulated by RsWRKY15-*RsPDR12* module in the radish taproots

Several strategies including minimizing HM accumulation, transport, and detoxification, have been investigated in plants. However, limited WRKY–ABCG/PDR interactions have demonstrated significant roles under Cd stress. WRKY TFs regulate gene expression under abiotic stress by binding to the W-box [(T)(T)TGAC(C/T)] in target gene promoters (Han et al. 2019). In *Arabidopsis*, AtWRKY13 directly targets *AtPDR8* to effectively decrease Cd accumulation (Sheng et al. 2019). AtPSE1 enhances Pb accumulation primarily by activating genes involved in PC synthesis and, to a lesser extent, by activating the transcription of the ABC transporter gene *AtPDR12*/*AtABCG40* (Fan et al. 2016). AtWRKY45 decreases Cd accumulation by activating the transcription of *AtPCS1* and *AtPCS2* in *Arabidopsis* (Li et al. 2023). *AtWRKY12* overexpression suppresses *AtGSH1* expression and increases Cd accumulation in *Arabidopsis* (Han et al. 2019). In soybean, GmWRKY142 decreases Cd uptake and accumulation by activating the transcription of *GmTCDT1*, *GmCDT1-1*, and *GmCDT1-2* (Cai et al. 2020). In wheat, *TaWRKY70* overexpression reduces Cd accumulation by positively regulating *TaCAT5* expression (Jia et al. 2021). While the WRKY–*PDR* module has been studied in several plant species, specific WRKY members vary among species (He et al. 2019; Sheng et al. 2019; Zhang et al. 2025). In radish, RsWRKY75 activates *RsPDR8* transcription by direct promoter binding, thereby reducing Cd accumulation in roots (Zhang et al. 2025). In *Arabidopsis*, AtWRKY13 similarly reduces Cd accumulation by activating *AtPDR8* expression (Sheng et al. 2019). In addition, AtWRKY33 activates *AtPDR12* expression, conferring tolerance to *Botrytis cinerea* in *Arabidopsis* (He et al. 2019). This study reveals that RsWRKY15 binds to the *RsPDR12* promoter and activates its transcription to reduce Cd accumulation in radish taproots (Fig. 5). The overexpression of *RsPDR12* and *RsWRKY15* decreased Cd accumulation in roots. Thus, the transcriptional regulatory network of the RsWRKY15–*RsPDR12* module positively regulates Cd uptake and accumulation in radish taproots. Based on these results, a hypothetical model of the RsWRKY15–*RsPDR12* module conferring reduced Cd uptake and accumulation is proposed (Fig. 9). Our study demonstrates that *NtPDR12* was upregulated in *RsWRKY15*-OE lines, corresponding with decreased Cd accumulation. Thus, the module is conserved across species, potentially facilitating the development of elite cultivars with low Cd accumulation. RsWRKY75 reduces Cd uptake and accumulation by activation of *RsPDR8* and *RsAPX1* transcription in radish (Zhang et al. 2025). Cd stress response typically involves multiple gene networks. For instance, NAC102 directly binds to the *WAKL1* promoter and induces its transcription, facilitating pectin degradation and reducing Cd binding (Han et al. 2023). These findings suggest that the RsWRKY15–*RsPDR12* module operates in conjunction with other regulatory networks under Cd stress, providing insight into the *RsPDR12*-mediated regulatory network in radish taproots under Cd stress.

## Materials and methods

### Plant materials, growth conditions and Cd treatments

The germinating seeds of a radish advanced inbred line ‘NAU-YH’ were cultivated in a 2:1 mixture of soil and vermiculite under controlled growth conditions with a photoperiod cycle of 14 h light at 25 °C and 10 h dark at 16 °C. Three-week-old radish seedlings were transferred to 1/2 Hoagland nutrient solution, which was renewed every 3 days (Xu et al. 2020). After 6 days, radish plants of uniform size were subjected to 0.1 mM CdCl_2_ treatment for 3, 6, 12, 24, 48 h and 7 days, with untreated plants serving as controls. Radish roots were harvested after Cd treatment, immediately frozen in liquid nitrogen, and stored at − 80 °C for subsequent analysis (Zhang et al. 2023b).

Seeds of WT (Col-0) and *RsPDR12*-OE *Arabidopsis* lines, along with WT and *RsWRKY15*-OE *N. benthamiana* lines were sterilized and cultivated on MS medium at 24 °C under CdCl_2_ treatment.

### RNA extraction and RT-qPCR analysis

Total RNA was extracted using a Total RNA Kit (Tiangen, Beijing, China). cDNA synthesis was performed with the HiScript II 1 st Strand cDNA Synthesis Kit (Vazyme, Nanjing, China). PCR was performed on a LightCycler® 480 System (Roche, Mannheim, Germany) (Xu et al. 2020). *RsActin* in radish, *AtActin* in *Arabidopsis*, or *NtActin* in *N. benthamiana* functioned as internal reference genes (Xu et al. 2012). Relative expression levels were determined by the 2^−ΔΔC*T*^ method with three biological and three technical replicates (Livak and Schmittgen 2001). All primers used in this study are listed in Supplementary Table S1.

### Promoter activity analysis of *RsPDR12 *and *RsWRKY15*

The upstream sequences of the ATG site were isolated as the promoter regions of *RsPDR12* and *RsWRKY15*. These promoters were fused with the pGreenII-0800-LUC and pCAMBIA1391-GUS vectors to produce the recombinant plasmids *pRsPDR12*::LUC, *pRsPDR12*::GUS, *pRsWRKY15*::LUC and *pRsWRKY15*::GUS. The plasmids were transformed into *Agrobacterium tumefaciens* strain GV3101 (pSoup) and infiltrated into *N. benthamiana* leaves. Plants were maintained under normal conditions for 2 days, then exposed to 0.27 mM CdCl_2_ for 8 h, and analyzed using a chemiluminescence imaging system (ChemiScope 6000, Clinx Science Instruments, China) and GUS staining (Wang et al. 2022). Relative LUC intensity was determined using a living fluorescence imager (Lb985, Berthold, Germany) (He et al. 2023a).

### Subcellular localization of RsPDR12 and RsWRKY15

The *RsPDR12* and *RsWRKY15* CDSs, excluding the stop codon, were inserted into the pCAMBIA1300-GFP vector to generate *35S::RsPDR12*::GFP and *35S::RsWRKY15*::GFP constructs. These were transformed into *A. tumefaciens* strain GV3101 and infiltrated into *N. benthamiana* leaves (Wang et al. 2022). PM-mCherry and pSuper::NF-YA4-mCherry proteins served as plasma membrane and nuclear markers, respectively (Yang et al 2022). GFP and mCherry signals were detected under a laser confocal microscope (LSM800, Zeiss, Germany) after 48 h (Dong et al. 2023).

### Analysis of Cd transport activity of *RsPDR12* in yeast

The *RsPDR12* CDS was inserted into the pYES2 vector. The empty vector and RsPDR12-pYES2 were transformed into yeast strain BY4741. The transformed yeast cells underwent serial 10 × dilutions and were spotted onto yeast nitrogen base (YNB)-Ura solid medium containing 0, 27, 55 and 82 μM CdCl_2_, with 2% glucose (expression suppressor) or 2% galactose (expression inducer). Plates were incubated at 30 °C for 3 days. To measure Cd content, yeast cells were cultured in YNB-Ura medium with 2% galactose for 20 h, then exposed to 0.27 mM CdCl_2_for 8 h. Cd content was determined using an inductively coupled plasma mass spectrometer (ICP-MS; NexION-5000G; Perkin Elmer, USA) (Zhang et al. 2023b; Gaddam et al. 2024).

### Ectopic transformation of *RsPDR12* and *RsWRKY15*

*A. tumefaciens* strain GV3101 containing *35S::RsPDR12* was introduced into *Arabidopsis* by the floral-dip method (Clough and Bent 1998), while *A. tumefaciens* strain GV3101 containing *35S::RsWRKY15* was introduced into *N. benthamiana* plants by the leaf-disc method (Wu et al. 2014). *RsPDR12*-OE *Arabidopsis* lines and *RsWRKY15*-OE *N. benthamiana* lines underwent hygromycin screening. Sterilized seeds of WT and two *RsPDR12*-OE *Arabidopsis* lines (OE-9 and OE-18) were cultivated on MS medium containing 50 μM CdCl_2_, while WT and two *RsWRKY15*-OE lines (OE-1 and OE-3) were cultivated on MS medium containing 55 μM CdCl_2_. For histochemical localization of Cd, *N. benthamiana* WT and *RsWRKY15*-OE plants were submerged in 1.56 mM dithizone dye solution for 6 h. The red–black Cd–dithizone precipitates were observed after washing plants with dH_2_O (Zhu et al. 2016). The Cd content of WT, *RsPDR12*-OE, and *RsWRKY15*-OE plants was determined using ICP-MS (NexION-5000G, Perkin Elmer, USA) (Xu et al. 2020; Liu et al. 2022).

### Cd^2+^ flux measurement in roots using a Cd-fluorescent probe and NMT

The net Cd^2+^ flux of WT, *RsPDR12*-OE (*RsPDR12*-OE-9 and *RsPDR12*-OE-18), and *RsWRKY15*-OE (*RsWRKY15*-OE-1 and *RsWRKY15*-OE-3) was measured using the Cd-fluorescent probe Leadmium™ Green AM (Invitrogen, Carlsbad, CA, USA). The plants were washed with distilled water, incubated with the Cd-fluorescent probe for 1 h, followed by washing with 0.9% NaCl solution. Signal detection was performed using a laser confocal microscope (LSM800, Zeiss, Germany) (Zhang et al. 2021; Gaddam et al. 2024).

The net Cd^2+^ flux of WT, *RsPDR12*-OE (*RsPDR12*-OE-9 and *RsPDR12*-OE-18), and *RsWRKY15*-OE (*RsWRKY15*-OE-1 and *RsWRKY15*-OE-3) was also measured using NMT (NMT100 Series, Younger USA LLC, Amherst, MA, USA) with iFluxes/imFluxes v.1.0. Microelectrodes were prepared using backfilling solution (10 mM Cd(NO_3_)_2_ + 0.1 mM KCl) and selective liquid ion-exchange cocktails (Wu et al. 2019; Tan et al. 2024). The microelectrodes were calibrated with 0.05, 0.1 and 0.5 mM Cd^2+^, and Cd^2+^ flux was measured under 0.1 mM Cd^2+^ treatment (Zhang et al. 2021).

### Transient transformation of *RsPDR12* and *RsWRKY15* in radish

*RsPDR12* and *RsWRKY15* CDSs were inserted into the pCAMBIA1300-GFP and pTCK303 vectors to produce *35S::RsPDR12*, RNAi::*RsPDR12*, *35S::RsWRKY15*, and RNAi::*RsWRKY15* constructs, which were introduced into *A. tumefaciens* strain GV3101. Two-week-old radish cotyledons underwent transient transformation (He et al. 2023b). Radish plants received CdCl_2_ treatment for 8 h, and H_2_O_2_ and O_2_^−^ levels were detected by DAB and NBT staining (He et al. 2023a). MDA and proline levels were measured according to a previous report (Thirumalaikumar et al. 2018).

### cDNA library screening and Y1H assay

The *RsPDR12* promoter was integrated into the pAbAi vector, and subsequently transferred into yeast strain Y1H Gold. The Y1H Gold containing pAbAi-*pRsPDR12* was used to screen the radish yeast library. Yeast cells were cultured on solidified dextrose (SD)/− Leu medium supplemented with 300 ng/mL AbA, followed by PCR detection and sequencing. The *RsWRKY15* CDS was inserted into the pB42AD vector, while the *RsPDR12* promoter sequences (510 bp upstream from the start codon), two truncated fragments (S1, − 510 to − 285 bp; S2, − 284 to − 1 bp) of the *RsPDR12* promoter, and the 3 × Motif (W1, − 222 to − 214 bp; W2, − 214 to − 206 bp; W3, − 150 to − 142 bp; W4, − 138 to − 130 bp) along with 3 × m-Motif (mW1, GAATCCCA) were incorporated into the pLacZi2μ vector. Both plasmids were co-transferred into yeast strain EGY48. Yeast cells of positive clones were transferred and cultured in SD/− Trp/− Ura medium containing X-gal at 30 °C for 2–3 days (Dong et al. 2022). The pB42AD and pLacZi2μ vectors were used as negative controls.

### Dual-luciferase reporter assay

The *RsPDR12* promoter was inserted into the pGreenII-0800-LUC vector to generate *pRsPDR12*::LUC. The *RsWRKY15* CDS was incorporated into the pCAMBIA1300-GFP vector to produce *35S::RsWRKY15*. *35S::RsWRKY15*, *pRsPDR12*::LUC, and the empty vector were transformed into *A. tumefaciens* strain GV3101 (pSoup) and introduced into *N. benthamiana* leaves. The infiltrated leaves were maintained under controlled growth conditions for 2 days and analyzed using a chemiluminescence imaging system (ChemiScope 6000, Clinx Science Instruments, China). The relative LUC intensity was determined using a living fluorescence imager (Lb985, Berthold, Germany) (Ying et al. 2023).

### Statistical analysis

Statistical analysis was performed using Duncan’s multiple comparison test (*P* < 0.05) or Student’s *t*-test using SPSS v.21.0 (IBM, New York, NY, USA). The results are presented as mean ± standard deviation of three biological replicates.

## Supplementary Information

Additional file 1: Supplementary Figure. S1 Amino acid sequence alignment of RsPDR12 and AtPDR12. At, Arabidopsis thaliana (AT1G15520). Supplementary Figure. S2 The upstream transcription factors of RsPDR12 were screened by yeast one-hybrid library. (A) Self-activation detection of RsPDR12 gene promoter. (B) Colony detection electrophoresis results via Y1H library screening with RsPDR12 promoter. Supplementary Figure. S3 Amino acid sequence alignment of RsWRKY15 and AtWRKY15. At, *Arabidopsis thaliana* (AT2G23320). Supplementary Figure. S4 The relative expression level of *RsPDR12* gene in OE-EV, RNAi-EV, *RsWRKY15*-OE, and *RsWRKY15*-RNAi radish cotyledons using the RT-qPCR analysis. Data are presented as the mean ± SD, *n* = 3. Bars with different lowercase letters are significantly different at *P* < 0.05. Supplementary Figure. S5 The Cd content in radish cotyledons under Cd treatment (0.27 mM CdCl_2_, 8 h). (A) The Cd content of EV, *RsPDR12*-OE and *RsPDR12*-RNAi under Cd treatment. (B) The Cd content of OE-EV, RNAi-EV, *RsWRKY15*-OE and *RsWRKY15*-RNAi under Cd treatment. Data are the mean ± SD of three replicates (t-test; ** *P* < 0.01; *** *P* < 0.001). Supplementary Figure. S6 The relative expression level of *NtPDR12* gene in WT and *RsWRKY15*-OE tobacco lines using the RT-qPCR analysis. Data are presented as the mean ± SD, *n* = 3. Bars with different lowercase letters are significantly different at *P* < 0.05.

Additional file 2: Supplementary Table 1. The list of primer pairs used in this study.

## Data Availability

The data will be available from the corresponding author upon reasonable request.

## Abbreviations

DLA: Dual-luciferase reporter assay
GFP: Green fluorescence protein
GUS: β-Glucuronidase
LUC: Firefly luciferase
NMT: Non-invasive micro-test technology
PDR: Pleiotropic drug resistance protein
ROS: Reactive oxygen species
TF: Transcription factor
Y1H: Yeast one-hybrid
