# Supplementary material for: RsWRKY15–RsPDR12 module regulates Cd uptake and accumulation by promoting Cd efflux in radish (Raphanus sativus L.)
Source: Mol Hortic. 2026 Feb 12;6:15. doi: 10.1186/s43897-025-00195-7 (PMC12896155; doi:10.1186/s43897-025-00195-7)
Supplement: Supplementary file 1 — Additional file 1: Supplementary Figure. S1 Amino acid sequence alignment of RsPDR12 and AtPDR12. At, Arabidopsis thaliana (AT1G15520). Supplementary Figure. S2 The upstream transcription factors of RsPDR12 were screened by yeast one-hybrid library. (A) Self-activation detection of RsPDR12 gene promoter. (B) Colony detection electrophoresis results via Y1H library screening with RsPDR12 promoter. Supplementary Figure. S3 Amino acid sequence alignment of RsWRKY15 and AtWRKY15. At, Arabidopsis thaliana (AT2G23320). Supplementary Figure. S4 The relative expression level of RsPDR12 gene in OE-EV, RNAi-EV, RsWRKY15-OE, and RsWRKY15-RNAi radish cotyledons using the RT-qPCR analysis. Data are presented as the mean ± SD, n = 3. Bars with different lowercase letters are significantly different at P < 0.05. Supplementary Figure. S5 The Cd content in radish cotyledons under Cd treatment (0.27 mM CdCl2, 8 h). (A) The Cd content of EV, RsPDR12-OE and RsPDR12-RNAi under Cd treatment. (B) The Cd content of OE-EV, RNAi-EV, RsWRKY15-OE and RsWRKY15-RNAi under Cd treatment. Data are the mean ± SD of three replicates (t-test; ** P < 0.01; *** P < 0.001). Supplementary Figure. S6 The relative expression level of NtPDR12 gene in WT and RsWRKY15-OE tobacco lines using the RT-qPCR analysis. Data are presented as the mean ± SD, n = 3. Bars with different lowercase letters are significantly different at P < 0.05. [file 43897_2025_195_MOESM1_ESM.zip › Figure-S1.pdf]

```
RsPDR12   MEGTSFQKASNSLRRDSSVWKRDSGMEIFSRSSREEDDEEALKWAALEKLPTFDRLRNGILTASHG...INEIDIEKLGFQDTKKLLERLIKVGEDEHEKLLWKLKDRIDRVGIDLPTIE   117
AtPDR12   MEGTSFHQASNSMRRNSSVWKKDSGREIFSRSSREEDDEEALRWAALEKLPTFDRLRKGILTASHAGGPINEIDIQKLGFQDTKKLLERLIKVGDDEHEKLLWKLKKRIDRVGIDLPTIE   120
Consensus megtsf  asns rr ssvwk dsg eifsrssreeddeeal waaleklptfdrlr giltash    ineidi klgfqdtkkllerlikvg dehekllwklk ridrvgidlptie

RsPDR12   VRFDHLKVEAEVHVGGRALPTFVNFMSNFVDKLLNSLHLLPNRKKKFTILNDVNGIVKPGRMALLLGPPSSGKTTLLLALAGKLDHELKETGRVSYNGHGMNEFVPQRAAAYIGQNDVHI   237
AtPDR12   VRFDHLKVEAEVHVGGRALPTFVNFISNFADKFLNTLHLVPNRKKKFTILNDVSGIVKPGRMALLLGPPSSGKTTLLLALAGKLDQELKQTGRVTYNGHGMNEFVPQRTAAYIGQNDVHI   240
Consensus vrfdhlkveaevhvggralptfvnf snf dk ln lhl pnrkkkftilndv givkpgrmalllgppssgkttlllalagkld elk tgrv ynghgmnefvpqr aayigqndvhi

RsPDR12   GEMTVRETFAYAARFQGVGSRYDMLTELARREKEANIKPDPDIDVFMKATSTAGEETNVMTDYILKILGLEVCADTMVGDDMLRGISGGQKKRVTTGEMLVGPSRALFMDEISTGLDSST   357
AtPDR12   GEMTVRETFAYAARFQGVGSRYDMLTELARREKEANIKPDPDIDIFMKAMSTAGEKTNVMTDYILKILGLEVCADTMVGDDMLRGISGGQKKRVTTGEMLVGPSRALFMDEISTGLDSST   360
Consensus gemtvretfayaarfqgvgsrydmltelarrekeanikpdpdid fmka stage tnvmtdyilkilglevcadtmvgddmlrgisggqkkrvttgemlvgpsralfmdeistgldsst

RsPDR12   TYQIVNSLRNYVHIFNGTALISLLQPAPETFNLFDDIFLIAEGEIIYEGPREHVVEFFETMGFKCPPRKGVADFLQEVTSKKDQMQYWSRPEEPYRFVRVREFAEAFQSFHVGRRLGDEL   477
AtPDR12   TYQIVNSLRNYVHIFNGTALISLLQPAPETFNLFDDIILIAEGEIIYEGPRDHVVEFFETMGFKCPPRKGVADFLQEVTSKKDQMQYWARRDEPYRFIRVREFAEAFQSFHVGRRIGDEL   480
Consensus tyqivnslrnyvhifngtalisllqpapetfnlfddi liaegeiiyegpr hvveffetmgfkcpprkgvadflqevtskkdqmqyw r  epyrf rvrefaeafqsfhvgrr gdel

RsPDR12   AVPFDKKKSHPAALTTKKFGVGIKELVNTSFSREYLLMKRNSFVYYFKFGQLLVMAFATMTLFFRTEMQKKTVIDGSLYTGALFFILMMLMFNGMSELSMTIAKLPVFYKQRDLLFYPAW   597
AtPDR12   ALPFDKTKSHPAALTTKKYGVGIKELVKTSFSREYLLMKRNSFVYYFKFGQLLVMAFLTMTLFFRTEMQKKTEVDGSLYTGALFFILMMLMFNGMSELSMTIAKLPVFYKQRDLLFYPAW   600
Consensus a pfdk kshpaalttkk gvgikelv tsfsreyllmkrnsfvyyfkfgqllvmaf tmtlffrtemqkkt  dgslytgalffilmmlmfngmselsmtiaklpvfykqrdllfypaw

RsPDR12   VYSLPPWLLKIPISFIEAALTTFITYYVIGFDPNIGRLFKQYILLVLMNQMASALFKMMAALGRNMIVANTFGAFSMLVFFALGGVVLSKDDIKKWWIWGYWISPIMYGQNAIVANEFFG   717
AtPDR12   VYSLPPWLLKIPISFMEAALTTFITYYVIGFDPNVGRLFKQYILLVLMNQMASALFKMVAALGRNMIVANTFGAFAMLVFFALGGVVLSRDDIKKWWIWGYWISPIMYGQNAILANEFFG   720
Consensus vyslppwllkipisf eaalttfityyvigfdpn grlfkqyillvlmnqmasalfkm aalgrnmivantfgaf mlvffalggvvls ddikkwwiwgywispimygqnai aneffg

RsPDR12   HSWSQAVPNSSDTLGVTVLKSRGFLPHAYWYWIGTGALLGFVVLFNFGFTLALTYLNSLGKPQAVLTEDPASNETEEL.....HVVDANANKKKGMVLPFEPHSITFDNVIYSVDMPQEM   832
AtPDR12   HSWSRAVENSSETLGVTFLKSRGFLPHAYWYWIGTGALLGFVVLFNFGFTLALTFLNSLGKPQAVIAEEPASDETELQSARSEGVVEAGANKKRGMVLPFEPHSITFDNVVYSVDMPQEM   840
Consensus hsws av nss tlgvt lksrgflphaywywigtgallgfvvlfnfgftlalt lnslgkpqav  e pas ete        vv a ankk gmvlpfephsitfdnv ysvdmpqem

RsPDR12   IEQGTQEDRLVLLKGVNGAFRPGVLTALMGVSGAGKTTLMDVLAGRKTGGYIDGNITISGYPKNQQTFARISGYCEQTDIHSPHVTVYESLVYSAWLRLPKEVDSNTRKMFIDEVMELVE   952
AtPDR12   IEQGTQEDRLVLLKGVNGAFRPGVLTALMGVSGAGKTTLMDVLAGRKTGGYIDGNITISGYPKNQQTFARISGYCEQTDIHSPHVTVYESLVYSAWLRLPKEVDKNKRKIFIEEVMELVE   960
Consensus ieqgtqedrlvllkgvngafrpgvltalmgvsgagkttlmdvlagrktggyidgnitisgypknqqtfarisgyceqtdihsphvtvyeslvysawlrlpkevd n rk fi evmelve

RsPDR12   LTPLRQALVGLPGESGLSTEQRKRLTIAVELVANPSIIFMDEPTSGLDARAAAIVMRTVRNTVDTGRTVVCTIHQPSIDIFEAFDELFLLKRGGEEIYVGPLGHESSHLINYFESIQGIS  1072
AtPDR12   LTPLRQALVGLPGESGLSTEQRKRLTIAVELVANPSIIFMDEPTSGLDARAAAIVMRTVRNTVDTGRTVVCTIHQPSIDIFEAFDELFLLKRGGEEIYVGPLGHESTHLINYFESIQGIN  1080
Consensus ltplrqalvglpgesglsteqrkrltiavelvanpsiifmdeptsgldaraaaivmrtvrntvdtgrtvvctihqpsidifeafdelfllkrggeeiyvgplghes hlinyfesiqgi

RsPDR12   KITEGYNPATWMLEVSTTSQEAALGVDFAQLYKNSELYKRNKELIKELSQPAPGSKDLYFPTQYSQSFWTQCMASLWKQHWSYWRNPPYTAVRFLFTIGIALMFGTMFWDLGGKTRTQQD  1192
AtPDR12   KITEGYNPATWMLEVSTTSQEAALGVDFAQVYKNSELYKRNKELIKELSQPAPGSKDLYFPTQYSQSFLTQCMASLWKQHWSYWRNPPYTAVRFLFTIGIALMFGTMFWDLGGKTKTRQD  1200
Consensus kitegynpatwmlevsttsqeaalgvdfaq yknselykrnkelikelsqpapgskdlyfptqysqsf tqcmaslwkqhwsywrnppytavrflftigialmfgtmfwdlggkt t qd

RsPDR12   LSNAMGSMYTAVLFLGLQNAASVQPVVNVERTVFYREQAAGMYSAMPYAFAQVFIEMPYVLVQAVVYGLIVYAMIGFEWTAAKFFWYLFFMYGSFLTFTFYGMMAVAMTPNHHIASVVSS  1312
AtPDR12   LSNAMGSMYTAVLFLGLQNAASVQPVVNVERTVFYREQAAGMYSAMPYAFAQVFIEIPYVLVQAIVYGLIVYAMIGFEWTAVKFFWYLFFMYGSFLTFTFYGMMAVAMTPNHHIASVVSS  1320
Consensus lsnamgsmytavlflglqnaasvqpvvnvertvfyreqaagmysampyafaqvfie pyvlvqa vyglivyamigfewta kffwylffmygsfltftfygmmavamtpnhhiasvvss

RsPDR12   AFYGIWNLFSGFLIPRPSMPVWWEWYYWLCPVSWTLYGLITSQFGDITEPMADG.TSVKQFIKDFYGFREGFLGVVAAMNVIFPLAFAIIFAIGIKSFNFQK                   1413
AtPDR12   AFYGIWNLFSGFLIPRPSMPVWWEWYYWLCPVAWTLYGLIASQFGDITEPMADSNMSVKQFIREFYGYREGFLGVVAAMNVIFPLLFAVIFAIGIKSFNFQK                   1422
Consensus afygiwnlfsgfliprpsmpvwwewyywlcpv wtlygli sqfgditepmad   svkqfi  fyg regflgvvaamnvifpl fa ifaigiksfnfqk
```
