# Supplementary material for: RsWRKY15–RsPDR12 module regulates Cd uptake and accumulation by promoting Cd efflux in radish (Raphanus sativus L.)
Source: Mol Hortic. 2026 Feb 12;6:15. doi: 10.1186/s43897-025-00195-7 (PMC12896155; doi:10.1186/s43897-025-00195-7)
Supplement: Supplementary file 1 — Additional file 1: Supplementary Figure. S1 Amino acid sequence alignment of RsPDR12 and AtPDR12. At, Arabidopsis thaliana (AT1G15520). Supplementary Figure. S2 The upstream transcription factors of RsPDR12 were screened by yeast one-hybrid library. (A) Self-activation detection of RsPDR12 gene promoter. (B) Colony detection electrophoresis results via Y1H library screening with RsPDR12 promoter. Supplementary Figure. S3 Amino acid sequence alignment of RsWRKY15 and AtWRKY15. At, Arabidopsis thaliana (AT2G23320). Supplementary Figure. S4 The relative expression level of RsPDR12 gene in OE-EV, RNAi-EV, RsWRKY15-OE, and RsWRKY15-RNAi radish cotyledons using the RT-qPCR analysis. Data are presented as the mean ± SD, n = 3. Bars with different lowercase letters are significantly different at P < 0.05. Supplementary Figure. S5 The Cd content in radish cotyledons under Cd treatment (0.27 mM CdCl2, 8 h). (A) The Cd content of EV, RsPDR12-OE and RsPDR12-RNAi under Cd treatment. (B) The Cd content of OE-EV, RNAi-EV, RsWRKY15-OE and RsWRKY15-RNAi under Cd treatment. Data are the mean ± SD of three replicates (t-test; ** P < 0.01; *** P < 0.001). Supplementary Figure. S6 The relative expression level of NtPDR12 gene in WT and RsWRKY15-OE tobacco lines using the RT-qPCR analysis. Data are presented as the mean ± SD, n = 3. Bars with different lowercase letters are significantly different at P < 0.05. [file 43897_2025_195_MOESM1_ESM.zip › Figure-S3.pdf]

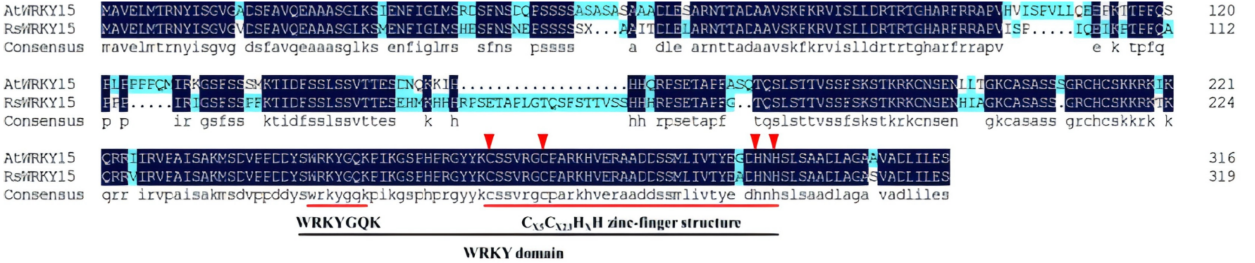
AtWRKY15
RsWRKY15
Consensus
120
112
221
224
316
319
WRKYGQK
CX5CX23HXH zinc-finger structure
WRKY domain
